# Supplementary material for: Automatic detection of methane emissions in multispectral satellite imagery using a vision transformer
Source: Nat Commun. 2024 May 14;15:3801. doi: 10.1038/s41467-024-47754-y (PMC11094139; doi:10.1038/s41467-024-47754-y)
Supplement: Supplementary file 1 — Supplementary text and figures [file 41467_2024_47754_MOESM1_ESM.pdf]

Supplementary for Automatic Detection of Methane Emissions in Multi-Spectral Satellite Imagery Using Transformers

Bertrand Rouet-Leduc[1,2*], Claudia Hulbert[2]
[1]DPRI, Kyoto University, Japan
[2]Geolabe, Los Alamos, New Mexico, USA
*To whom correspondence should be addressed;
E-mail: rouetleduc.bertrand.5s@kyoto-u.ac.jp.

Synthetic test data distribution

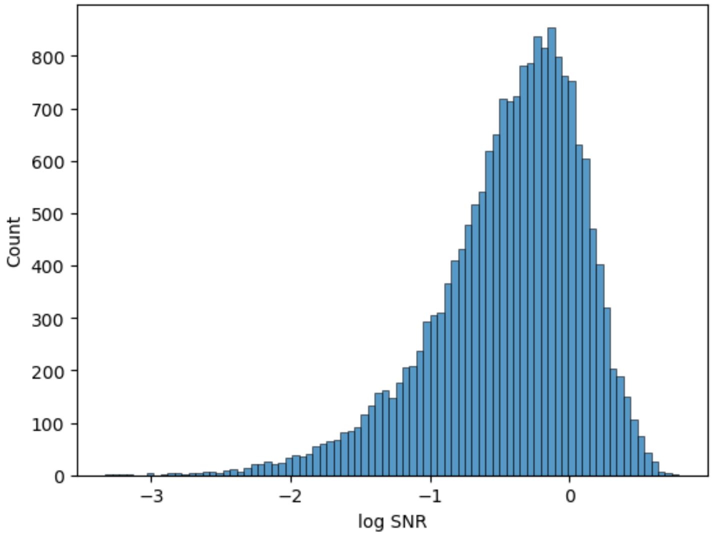

Figure S1: Distribution of the signal to noise ratio of the samples used for testing the model on synthetic data in Fig. 2 of the main text.

Fig. S1 shows the signal-to-noise ratio distribution of the approx. 40 000 synthetic test samples used in Fig. 2 of the main text. As shown in Fig. 2 of the main text, most of the missed

detections of our model are for signal to noise ratios below 5%, in the left tail of the distribution in Fig S1. The signal-to-noise distribution is not heavy tailed in the high values of SNR because highly concentrated methane plumes strongly absorb in band 12, so that the methane signal we are looking for is bound (by B12 at 0 reflectance).

Plume rate versus plume extent in the Carbon Mapper catalogue

Fig. S2 shows the empirical distribution of plume leak rate versus plume extent in the Carbon Mapper dataset, using the plume mask of highest concentration. We use a negative exponential to fit this empirical relationship (shown as a dark line). The results of applying our method to the Carbon Mapper dataset have a clearer trend when plot against the catalogued plume extent, and we use this fit for the secondary axis in Fig. 3 of the main text.

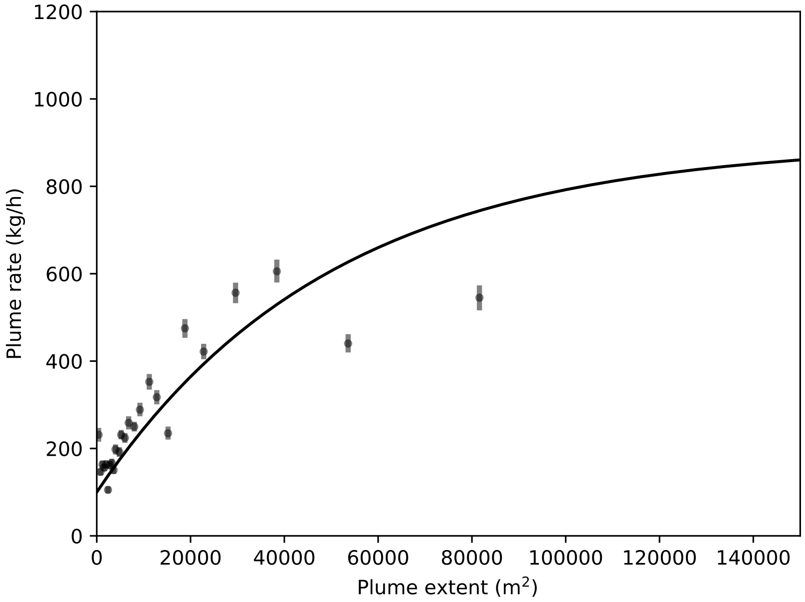

Figure S2: Distribution of plume leak rate versus plume mask extent in the Carbon Mapper database. The plume mask's extent is that of the highest concentration mask from the database. The black line shows the fit of plume rate versus plume extent used as the secondary x axis in Fig. 3 of the main text.

Application of our model to methane controlled release experiments

To further validate our algorithm, we analyze Sentinel-2 data timed with controlled methane release experiments described in a recent study.[1] 82 controlled methane releases were conducted over two months, and timed with satellite overpasses for various American, European, and Chinese satellites. Four releases of respective leak rates of 1.1, 1.2, 1.5 and 1.6 tons/h were timed with Sentinel-2 overpasses and analyzed by various teams.

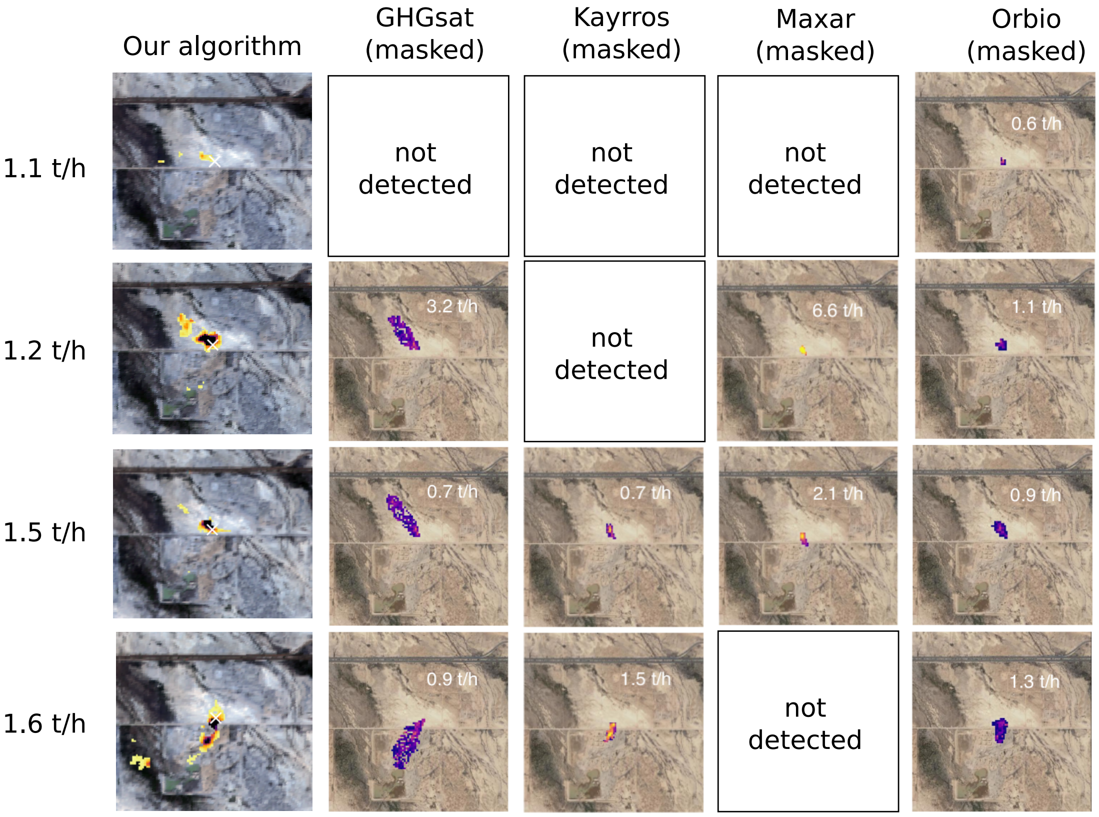

Figure S3: Application of our algorithm to four methane controlled releases analyzed in a recent study,[1] and comparison with masked detections made in Sentinel-2 by the companies participating in the blind test. In contrast to these groups, human analysts are not involved, and neither the wind nor the location of the controlled release (white cross) are known to our deep learning model (they are only indicated for reference). Fig. adapted from.[1]

Fig. S3 shows the application of our model to these four respective releases, compared to the masked detections made by the companies participating to the blind test. Our model is able to successfully detect all four releases, with very little false positives outside of the associated plumes. Note that pre-masking methane retrieval fields published by the various teams in this

study[1] all contain a large number of false positives pixel-wise (including for more precise, task-modes satellites), which further illustrate the much cleaner output and degree of automation using our algorithm. Unmasked retrieval fields for the Sentinel-2 data analyzed here are shown in Fig. S4.

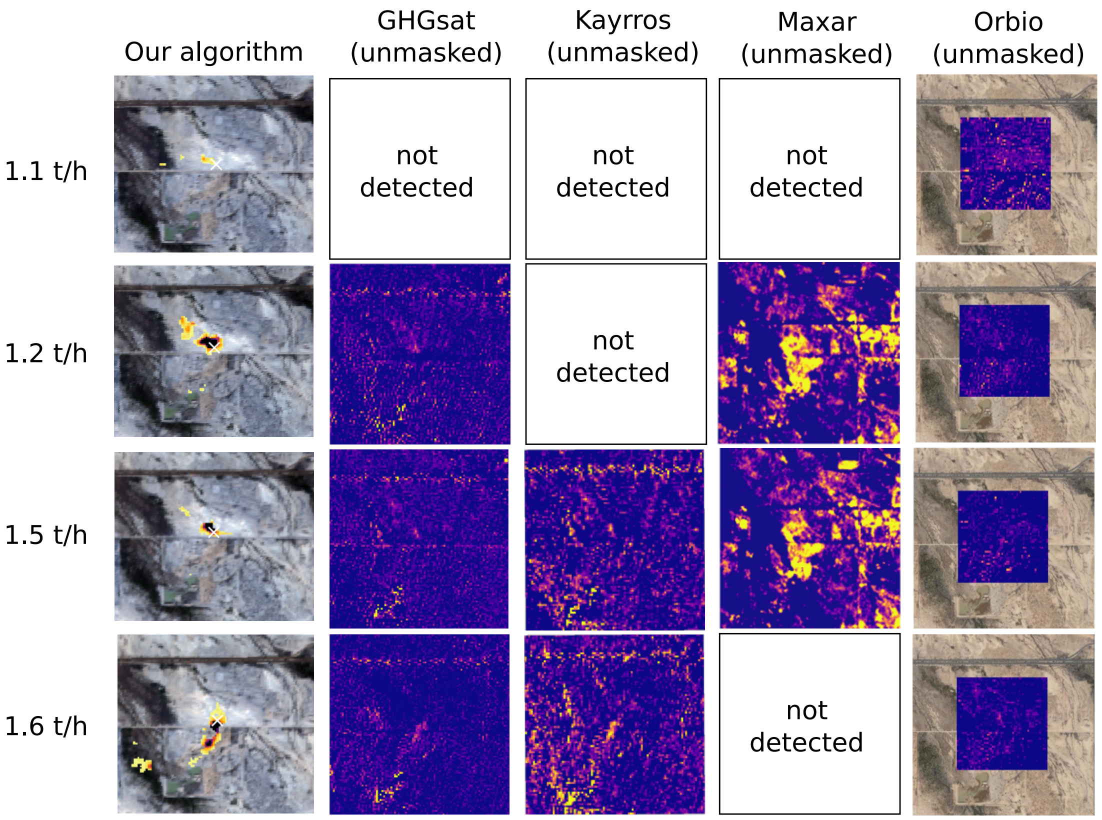

Figure S4: Same application of our algorithm to four methane controlled releases, and comparison with unmasked methane retrieval fields in Sentinel-2 computed by the companies participating in the blind test. This illustrates the number of false positives in unmasked methane retrieval fields. Note that plume detection involves analysis by a human operator. Fig. adapted from.[1]

Numerical experiments on the deep learning model's behavior

Figure S5 analyzes the distribution of the model's detections on examples from the test set. The figure shows the ratio of the spatial distribution of the generated ground truth to the spatial distribution of the model's output. Because this ratio is flat (except at the very edges), this shows that the model is not biased in detecting plumes in the center of its input.

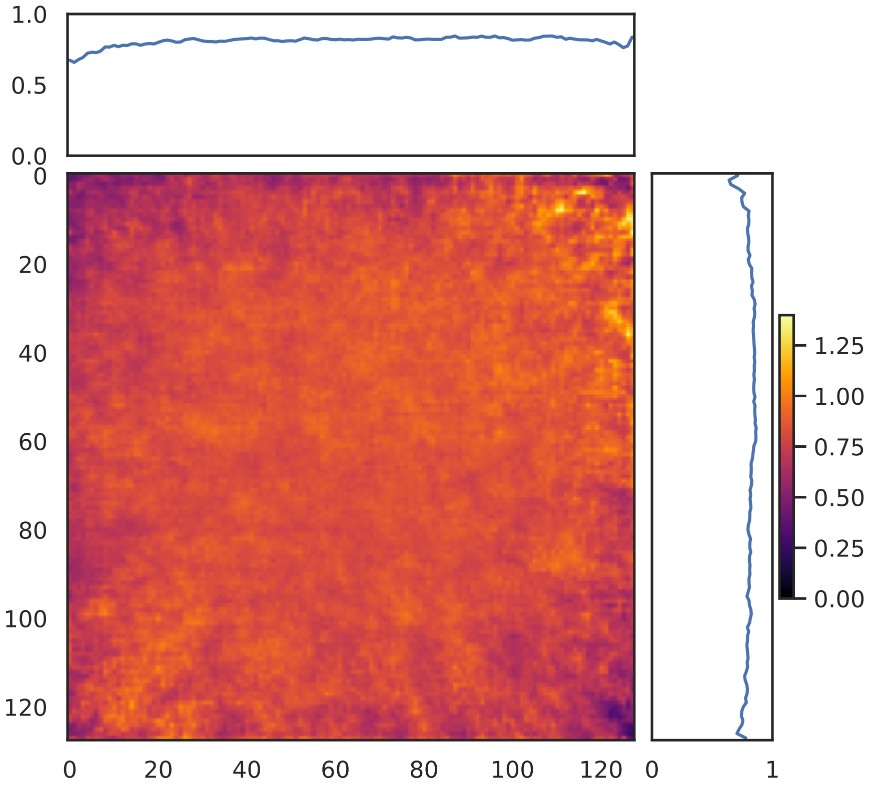

Figure S5: Spatial distribution of the neural network detection capabilities. The figure shows the ratio of the distribution of synthetic plumes (on the test set tiles over Argentina and Afghanistan) to the distribution of the neural network's detections, computed from 12,000 samples. The figure shows that the neural network's response is mostly flat, except close to the edges, showing that there is no bias in favor of detecting a plume at the center of the input. In particular, by putting the Carbon Mapper cataloged plumes at the center of the neural network's input, such as in Figures 3 and 4 of the main text, they are not more likely to be detected.

Performance of a CNN model

Figure S6 shows the performance of a CNN model trained on the same data as the transformer model used in the main text. As the transformer model, the CNN vastly outperforms the MBMP method, but suffers from noticeably more false positives than the transformer model.

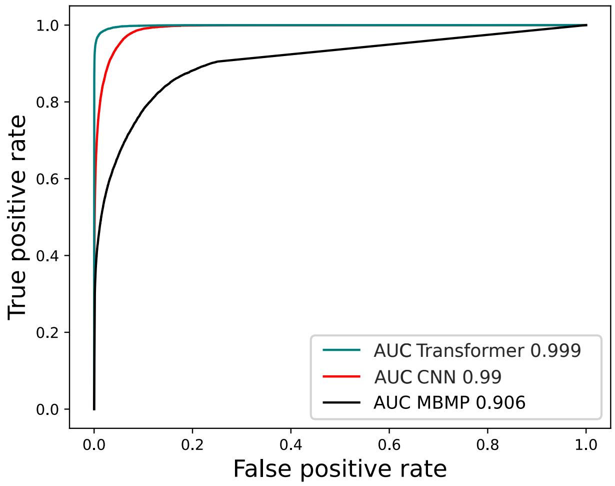

Figure S6: Performance of a fully convolutional autoencoder (in red) versus the unet transformer model evaluated in the main text (in blue) and the MBMP method (in black), in terms of area under the ROC curve, calculated on 10 000 samples from the test set. The transformer model outperforms the CNN, especially in terms of false positive rate.

Sampling of training, validation and test data

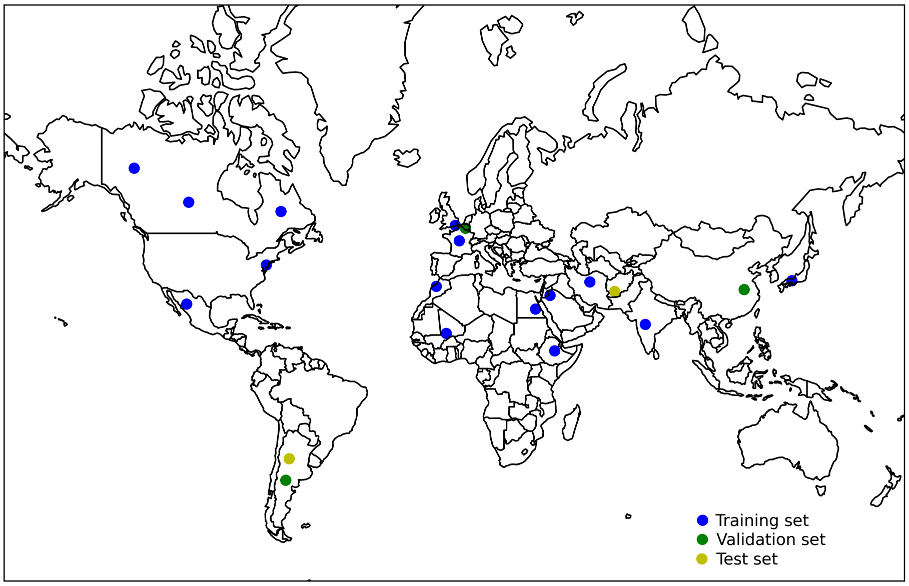

Figure S7: Spatial regions used for downloading Sentinel-2 data in this study. The database is sampled from different regions in order to improve the robustness of the model.

Figure S7 shows the distribution of the tiles used to create a database of Sentinel 2 data in which we embed synthetic plumes, to train, validate and test the deep learning model tasked with finding methane plumes.

Deep detections of catalogued plumes

Figure S8 shows the fraction of catalogued Carbon Mapper leaks that were detected by our deep learning model, as a function of leak rate. Compared with Fig. 3 of the main text, we can see that the main factor explaining detection is the plume's extent, rather than the plume's leak rate. However, plume extent and plume rate are closely correlated, with variations mostly due to wind conditions, and the plume's extent can be rescaled as an expected plume rate (using the fit from Fig. S1 of the supplementary), as shown in the secondary axis of Fig. 3 of the main text.

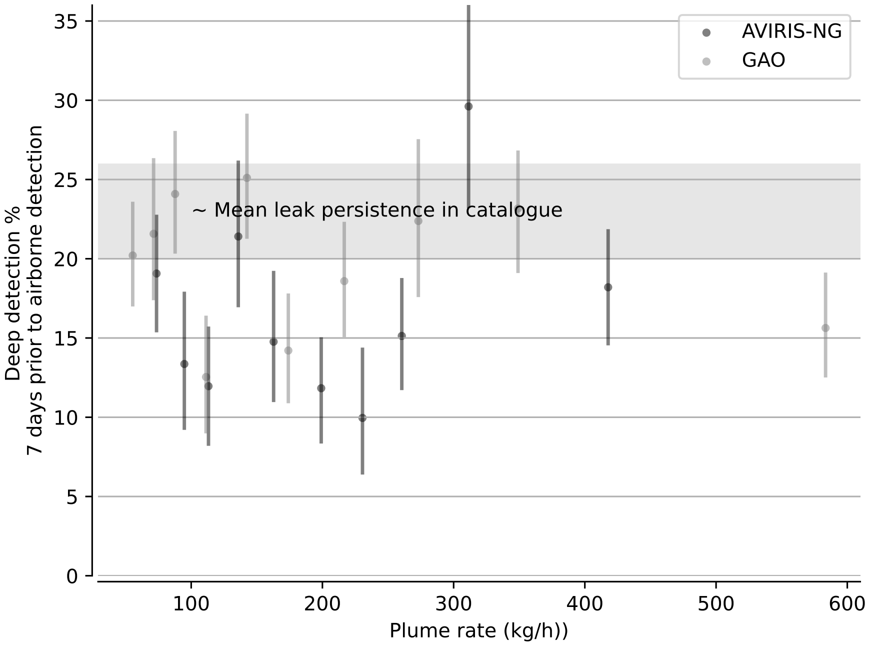

Figure S8: Fraction of catalogued methane leaks detected by our deep learning model as a function of (non-rescaled) leak rate. As in Fig. 3 of the main text, but showing detection rate as a function of leak rate instead of plume extent.

Supplementary References

[1] Sherwin, E. D. *et al.* Single-blind test of nine methane-sensing satellite systems from three continents. *EarthArXiv* (2023).
